# Evaluation of Permeation Enhancers for Vaginal Delivery of Buserelin Acetate Using a Validated Chromatographic Method and Ex Vivo Porcine Model

**DOI:** 10.3390/pharmaceutics17091181

**Published:** 2025-09-11

**Authors:** AHM Musleh Uddin, Roy N. Kirkwood, Kiro R. Petrovski, Souha H. Youssef, Baljinder Singh, Songhita Mukhopadhyay, Yunmei Song, Sanjay Garg

**Affiliations:** 1 School of Animal and Veterinary Sciences, Adelaide University, Roseworthy Campus, Roseworthy, SA 5371, Australia; ahm.uddin@adelaide.edu.au (A.M.U.); roy.kirkwood@adelaide.edu.au (R.N.K.); kiro.petrovski@adelaide.edu.au (K.R.P.); 2 Centre for Pharmaceutical Innovation (CPI), College of Health Sciences, Adelaide University, Adelaide, SA 5000, Australia; souha.youssef@unisa.edu.au (S.H.Y.); baljinder.singh@mymail.unisa.edu.au (B.S.); songhita.mukhopadhyay@mymail.unisa.edu.au (S.M.); may.song@unisa.edu.au (Y.S.); 3 Davies Livestock Research Centre, Roseworthy Campus, Roseworthy, SA 5371, Australia

**Keywords:** HPLC, peptide drugs, vaginal mucosa, Franz cells, permeation coefficient, kinetic analysis

## Abstract

**Background/Objectives**: This study aimed to enhance the vaginal permeation of buserelin acetate (BA), a synthetic gonadotropin-releasing hormone (GnRH) analogue, by evaluating various permeation enhancers (PEs) using a validated reversed-phase high-performance liquid chromatography (RP-HPLC) method and an ex vivo porcine vaginal model. **Methods**: A robust RP-HPLC method was developed and validated according to ICH Q2 (R2) guidelines to enable accurate quantification of BA in permeation samples. The analytical method demonstrated high specificity, linearity (R^2^ = 0.9999), accuracy (98–102%), precision (%RSD < 2%), robustness, and stability. Using this method, ex vivo permeation studies were conducted with six different PEs: 2-hydroxypropyl-β-cyclodextrin, sodium dodecyl sulfate, poloxamer 188, Span 80, Tween 80, and chitosan. **Results**: Among all tested PEs, chitosan demonstrated the best enhancement of BA permeation. It achieved the highest flux (J) (0.64 ± 0.03 × 10^−2^ µg/cm^2^·h) and apparent permeability coefficient (P_app_) (16.20 ± 0.84 × 10^−5^ cm/h), both of which were statistically significantly higher (*p* < 0.05) than those of all other enhancer groups. Kinetic modelling indicated a non-Fickian, biphasic permeation mechanism best described by the Makoid–Banakar model. **Conclusions**: These findings highlight chitosan’s potential as an effective intravaginal delivery vehicle for peptide therapeutics and establish the validated HPLC method as a reliable platform for future formulation development and translational studies in mucosal drug delivery.

## 1. Introduction

Buserelin acetate (BA) is a synthetic analogue of gonadotropin-releasing hormone (GnRH) widely utilized in veterinary medicine for managing reproductive disorders, including ovarian cysts and synchronization of ovulation [1,2]. The efficacy of BA is hindered by limited permeability and enzymatic degradation, especially through non-invasive mucosal routes, where the epithelium poses both physical and metabolic barriers to drug absorption [3,4]. Permeation testing provides valuable insights into peptide permeability, enabling better prediction of clinical outcomes and optimization of drug delivery strategies. Consequently, permeation enhancers (PEs) are essential to overcome these limitations and enable more effective intravaginal BA delivery [3,4].

Vaginal permeation studies are considered the gold standard for evaluating and optimising drug delivery systems intended for vaginal route administration, as they closely simulate in vivo conditions and provide realistic estimates of drug absorption, permeation, and bioavailability [5,6]. This approach preserves the tissue’s complex bilayer architecture comprising the epithelium and stroma, along with its enzymatic activity and barrier functionality, thereby providing physiologically relevant data on permeation kinetics and formulation performance that cannot be replicated by synthetic membranes or cell monolayers. Porcine vaginal mucosa, in particular, demonstrates exceptional structural and biochemical homology to human tissue in thickness, lipid composition, and tight junction density, making it a validated surrogate for human vaginal absorption studies [6,7].

The current literature focuses predominantly on injectable or nasal BA formulations, with scant research on intravaginal systems [8,9]. While several PEs show promise for enhancing mucosal delivery, their comparative efficacy for vaginal peptide permeation remains unexplored. Cyclodextrins enhance solubility and membrane fluidity by extracting lipid components [10]; surfactants like Sodium dodecyl sulfate (SDS) disrupt epithelial integrity via lipid bilayer intercalation [11], while non-ionic agents (Span 80, Tween 80) reduce mucus viscosity and open tight junctions [12]. Poloxamer 188 modulates membrane fluidity and prolongs mucosal contact [13], and chitosan enhances paracellular transport via tight junction opening and mucoadhesion [14].

The therapeutic effectiveness of BA largely depends on accurate and reliable quantification methodologies [15], but traditional analytical approaches often face limitations regarding sensitivity, specificity, and reproducibility, necessitating the development of new and reliable chromatographic methods [16]. Reversed-phase high-performance liquid chromatography (RP-HPLC) has become the choice for separations and analysis of peptide-based pharmaceuticals due to its efficiency, selectivity, and suitability for hydrophilic and hydrophobic analytes [17]. Peptide drugs like goserelin, buserelin, and triptorelin present unique analytical challenges due to their polarity, susceptibility to degradation, and the tendency for peak tailing [18,19]. Thus, the chromatographic conditions must be meticulously optimised and utilise suitable mobile phases, column chemistries, and detection wavelengths to achieve robust and high-quality analytical performance [20]. Ex vivo permeation studies with PEs are crucial for understanding drug absorption, quantifying permeability coefficients, and assessing formulation performance. These comparisons provide essential preliminary data for improving formulations and subsequently designing animal trials to evaluate safety, pharmacokinetics, and therapeutic efficacy [21].

This study aimed to develop and validate a simple and reliable RP-HPLC method for the determination of BA, adhering to the guidelines outlined by the International Council for Harmonisation (ICH) Q2 (R2) [22]. Subsequently, the validated method was utilised to evaluate the permeation characteristics of BA with PEs through porcine vaginal mucosa, utilizing kinetic modelling to illustrate the primary drug release mechanisms. These findings not only highlight the potential of chitosan-based systems for vaginal peptide delivery but could also inform the design of intravaginal BA formulations, offering a novel pathway to improved therapeutic outcomes in veterinary reproduction.

## 2. Materials and Methods

### 2.1. Instrumentation and Chromatographic Analysis Software

The Shimadzu^®^ LC system (Shimadzu Corporation, Kyoto, Japan) was utilized for the analysis, featuring an LC-20ADXR pump, DGU-20A3 degasser, SIL-20A HT autosampler, and an SPD-M20A photodiode array detector (PDA). A Phenomenex column Luna series (100 Å, 250 × 4.6 mm, 5 µm) was used as the stationary phase. A bath sonicator was used to degas the mobile phase (MP). For the analytical procedures, a calibrated pH meter and a digital weighing balance (AB204-S; Mettler Toledo, Greifensee, Switzerland) were employed. All HPLC data acquisition and processing were performed using LabSolutions software (version 5.96) (Shimadzu Corporation, Kyoto, Japan).

### 2.2. Chemicals and Solvents

#### Pure Samples, Reagents and Solvents

The BA and acetonitrile (HPLC grade) were purchased from Sigma-Aldrich (Castle Hill, NSW, Australia). Milli-Q (MQ) water (18.2 mΩ) was obtained using a Sartorius water purification system (Millipore Milli-Q, Darmstadt, Germany). Cellulose acetate filters with a 0.45 μm pore size were sourced from Sartorius (Göttingen, Germany). Trifluoroacetic acid (TFA) (Synthesis grade) was purchased from Scharlau (Barcelona, Spain). The PEs: 2-hydroxypropyl-β-cyclodextrin, sodium dodecyl sulfate (SDS), Poloxamer 188, Span 80, Tween 80, and chitosan (low molecular weight, deacetylated) were all purchased from Sigma-Aldrich (Castle Hill, NSW, Australia).

### 2.3. Method Development and Validations

#### Chromatographic Analysis

The chromatographic conditions used for the quantification of BA were as follows: a stationary phase Phenomenex C18 column and an isocratic mode of MP composed of water and acetonitrile (70:30, *v*/*v*) with 0.035% TFA, adjusted to pH 2.5. A flow rate of 0.8 mL/min and an injection volume of 10 μL were applied. The wavelength of 220 nm for the detection of BA peaks was selected. Chromatographic analysis was conducted at a column temperature of 30 °C.

### 2.4. Preparation of Standard Stock Solution

Briefly, 1.00 mg of BA was dissolved in MQ water, and the volume was made up to 25.00 mL in a volumetric flask to obtain a stock solution with a concentration of 40.00 μg/mL. Serial dilutions were then carried out using MQ water to prepare the working solutions.

### 2.5. Method Validation

The developed RP-HPLC method was validated as per ICH Q2 (R2) guidelines [22]. The parameters of specificity, system suitability, linearity and range, accuracy, precision, and robustness were evaluated.

### 2.6. Specificity and Peak Purity

Specificity is the ability to test the analyte unambiguously in the presence of anticipated components. The specificity of the procedure was confirmed by evaluating any interference from blank and working solutions at the retention time (Rt) of the analyte. In this study, a standard stock solution of BA (40.00 μg/mL) was used to prepare a working solution of 15.00 μg/mL. Additionally, peak purity was assessed using a photodiode array detector (PDA) by comparing UV spectra at the leading edge, apex, and trailing edge of the BA peak. The purity index was >0.999, indicating that the BA peak was spectrally homogeneous and free from co-eluting impurities.

### 2.7. System Suitability

The system suitability of BA was evaluated using a working solution of 15.00 μg/mL (*n* = 6). The parameters tailing factor, number of theoretical plates, resolution, and relative standard deviation (RSD) of the peak area were assessed to ensure the adequacy of the chromatographic system.

#### 2.7.1. Linearity and Range

From the primary BA stock solution, a series of 13 concentrations (0.05, 0.10, 0.20, 0.40, 0.60, 0.80, 1.00, 5.00, 10.00, 15.00, 20.00, 25.00, and 30.00 µg/mL) were prepared by serial dilution using the same diluent. The mean peak area obtained from RP-HPLC analysis was plotted against each corresponding concentration to construct the calibration curve. Linearity was assessed by calculating the regression equation and squared correlation coefficient (R^2^). The RSD of the response factor across the concentration levels was also calculated to confirm the precision and consistency of the method across the established range.

#### 2.7.2. Accuracy

Accuracy was evaluated by performing recovery studies at three BA concentration levels (1.00 μg/mL, 15.00 μg/mL, and 30.00 μg/mL), and each level was prepared using the standard stock solution and MQ as the diluent. The samples were analysed, and the results were expressed as mean recovery (%) ± RSD.

#### 2.7.3. Precision

The precision of the method was assessed through intra-day and inter-day variation studies. Three BS concentrations (1.00 μg/mL, 15.00 μg/mL, and 30.00 μg/mL) were analysed for precision analysis. Each concentration was injected six times at different intervals within the same day to determine intra-day precision, and the procedure was repeated on three consecutive days to determine inter-day precision. The results were reported as mean recovery (%) ± RSD. The RSD value of less than 2.00% was considered acceptable, indicating the method’s repeatability and reproducibility.

#### 2.7.4. LOD and LOQ

The limit of detection (LOD) and limit of quantification (LOQ) serve as measures of the analytical method’s sensitivity. For BA, these values were determined using the signal-to-noise ratio obtained from the RP-HPLC chromatogram [23]. LOD and LOQ corresponded to signal-to-noise ratios of 3:1 and 10:1, respectively.

#### 2.7.5. Robustness

To assess the robustness of the developed RP-HPLC method, deliberate variations were introduced into key chromatographic parameters to evaluate the method’s resilience under minor changes. The tested parameters included column temperature (25 °C, 30 °C, and 35 °C), mobile phase composition (water:acetonitrile ratios of 68:32, 70:30, and 72:28 *v*/*v*), and flow rate (0.70, 0.80, and 0.90 mL/min). Three replicate injections were performed for each variation, and RSD values below 2% across all tested conditions confirmed the method’s robustness and reliability.

#### 2.7.6. Stability Test

The stability of BA standard solutions was assessed over 28 days under refrigerated conditions (4 °C) to ensure samples remained intact during sample preparation, short-refrigerated holding, and HPLC analysis. The samples were analysed at four time points: Day 1, Day 7, Day 14, and Day 28. Stability was evaluated by calculating the mean recovery (%) and RSD at each interval, to determine any significant changes in analyte concentration over time. Our study does not establish BA shelf-life or characterise degradation pathways; therefore, dosage form stability and forced-degradation (stress) studies were not included in this study.

### 2.8. Ex Vivo Permeation Studies

#### 2.8.1. Tissue Preparation

Fresh porcine vaginal tissue was collected from a local slaughterhouse (Big River Pork, Murray Bridge, South Australia, Australia) after the scavenged tissue collection approval and immediately transported to the laboratory in phosphate-buffered saline (pH 7.40). The mucosal layer was carefully separated from the underlying connective tissue using blunt dissection and stored at −20 °C in aluminium foil for use. 

#### 2.8.2. Franz Diffusion Cell Setup

**Figure 1:**
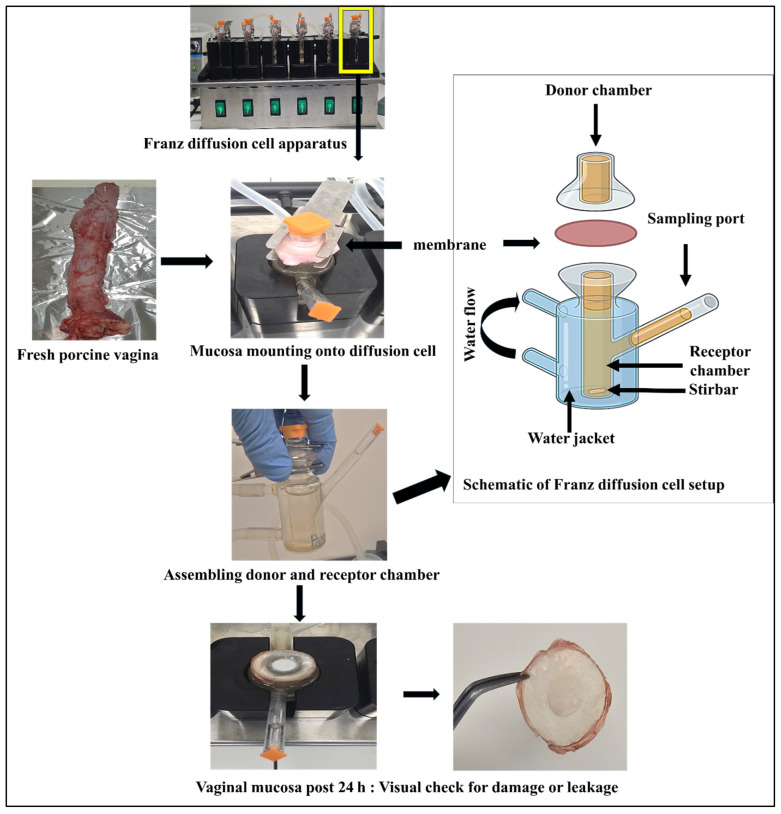
Workflow of the ex vivo permeation study of buserelin acetate (BA) using porcine vaginal mucosa in a Franz diffusion cell setup.

Permeation experiments were performed using vertical Franz diffusion cells with a diffusion area of 0.065 cm^2^ (Figure 1). The receptor compartment had a volume of 5.00 mL and was filled with simulated porcine vaginal fluid (SPVF) (pH 6.80) maintained at 37.00 ± 0.5 °C to mimic physiological conditions. The mucosal membrane was mounted between the donor and receptor compartments, ensuring the mucosal side faced the donor. The donor compartment contained 1.00 mL of the test sample. The receptor medium was continuously stirred using a magnetic stirrer to maintain uniformity and prevent concentration gradients.

To evaluate the impact of PEs on BA permeation, six experimental groups were established (Table 1). Each formulation consisted of BA combined with 10% (*w*/*w*) of a selected enhancer: 2-hydroxypropyl-β-cyclodextrin, Poloxamer 188, Span 80, Tween 80, or chitosan and 0.1% (*w*/*w*) sodium dodecyl sulfate (SDS), all calculated relative to the BA content. BA concentration was 40.00 µg/mL, which was consistently employed across the following seven test groups.

Each formulation (1.00 mL) was placed in the donor compartment, and all tests were conducted under identical conditions.

### 2.9. Sample Collection

Samples were withdrawn from the receptor compartment at the predetermined time intervals of 1, 2, 3, 4, 5, 6, 8 and 24 h. At each time point, 200 µL was removed and immediately replaced with an equal volume of fresh SPVF. All samples were analysed using a validated RP-HPLC method to quantify the amount of BA permeated.

### 2.10. Apparent Permeability Coefficient (P_app_)

The apparent permeability coefficient quantifies how readily a compound permeates through a biological membrane [24,25] and is calculated as

P_app_ = J/C_0_ (1)

where:

P_app_ is in cm/s

J is the flux rate (µg/cm^2^/h)

C_0_ is the initial drug concentration in the donor compartment (µg/mL)

Flux (J) was calculated by dividing the slope obtained by plotting the cumulative amount of BA permeated through the porcine vaginal mucosa versus time (t, 0–24 h) with the effective diffusion area (A) exposed to the drug.

### 2.11. Kinetic Analysis

The release profiles of BA during the ex vivo permeation studies were analysed by fitting the cumulative permeation data to several mathematical kinetic models [26]. The quality of fit for each model was determined by calculating the adjusted coefficient of determination (R^2^ adjusted), the Akaike Information Criterion (AIC), and the model selection criterion (MSC). These model-fitting analyses aimed to identify the most suitable mathematical representation of the drug’s permeation kinetics through the porcine vaginal mucosa.

### 2.12. Statistical Analysis

Linear regression analyses, including calibration curve construction and parameter calculations, were performed to evaluate method linearity and quantification accuracy. Statistical evaluation of J and P_app_ was conducted to assess the effect of various PEs on the vaginal permeation of BA. Data from ex vivo permeation studies were collected in triplicate (*n* = 3 per group), and values are presented as mean ± standard error (SE). A one-way analysis of variance (ANOVA) was performed to determine whether statistically significant differences existed among the enhancer groups for both J and P_app_. Upon finding significant group effects (*p* < 0.05), Tukey’s Honest Significant Difference (HSD) post hoc test was employed to conduct pairwise comparisons between the groups. All statistical analyses and graphical representations were carried out using OriginLab Pro 2025 (OriginLab Corporation, Northampton, MA, USA). Kinetic modelling of the ex vivo permeation data was conducted using DDSolver (an Excel-based add-in program). The quality of fit was evaluated based on adjusted R^2^, AIC, and MSC, ensuring a robust interpretation of the permeation kinetics.

## 3. Results

### 3.1. Chromatographic Conditions and Chromatograms

**Figure 2:**
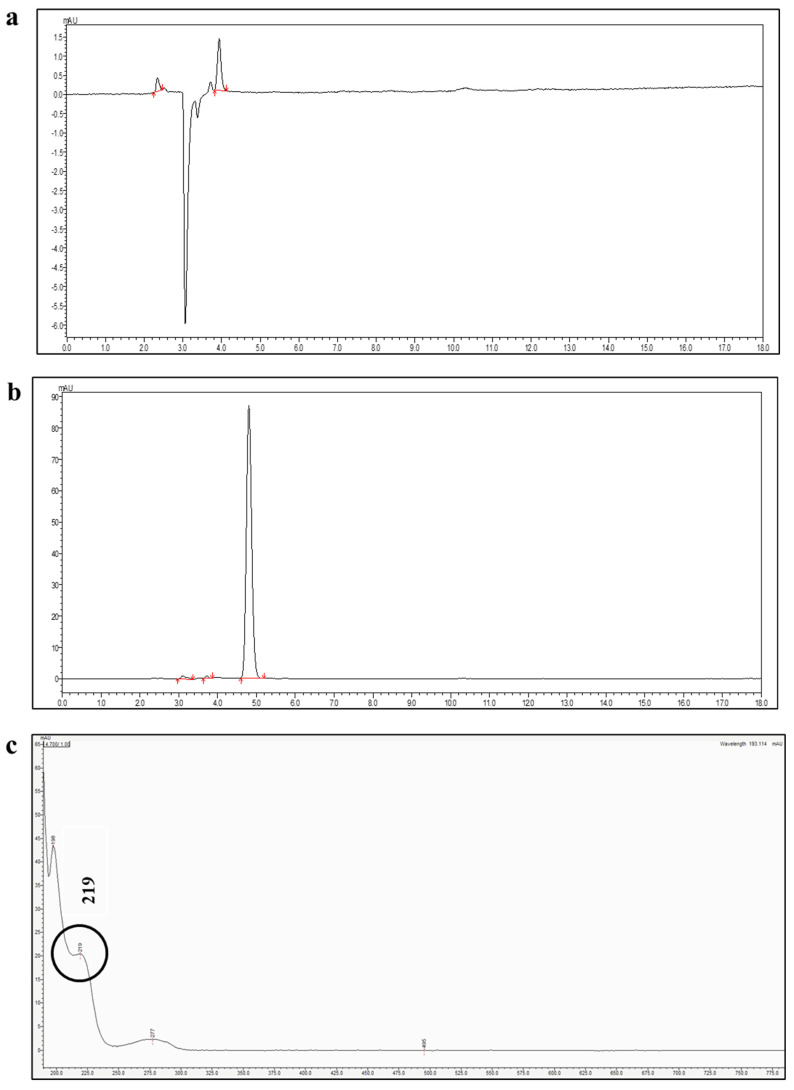
HPLC chromatogram and analysis of buserelin acetate (BA) for specificity and purity studies: (**a**) Blank sample, (**b**) BA sample, and (**c**) UV spectrum of BA, the circle in the UV spectrum highlights the absorption of the analyte, which was selected as the detection wavelength for the HPLC analysis of BA.

Buserelin acetate (BA) was successfully quantified using a validated RP-HPLC method with UV detection at 220 nm. Under the optimized conditions, BA exhibited a sharp, symmetric peak with minimal tailing and no baseline interference, indicating good chromatographic performance (Figure 2). The retention time was consistent across multiple injections. The selected MP composition was used as the diluent to maintain consistency and prevent compatibility or baseline shift issues during analysis. No interfering peaks from excipients were observed, confirming the specificity and efficiency of the method.

### 3.2. Method Validation

#### 3.2.1. Specificity

**Figure 3:**
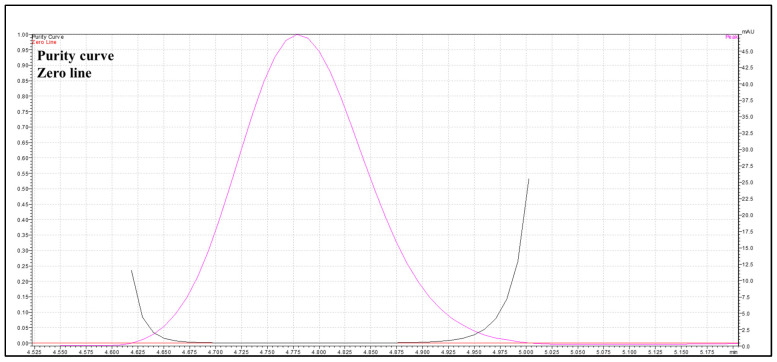
Peak purity curve of buserelin acetate (BA) using a photodiode array detector. Magenta curve represents the purity curve of the BA, the black line represents the zero line for comparison.

The resulting peak purity index was 1.0000, confirming compliance with established acceptance criteria [20]. Furthermore, the overall purity index exceeded the corresponding single-point threshold (Figure 3). As shown in the chromatograms (Figure 2), no interfering peaks were detected at the analyte retention times.

#### 3.2.2. System Suitability

The %RSD values for all key chromatographic parameters, including peak area and retention time, were found to be below 2%, based on six replicate injections of the working standard solution, indicating excellent consistency of the system. All results met the established acceptance criteria, including %RSD < 2%, theoretical plate number >2000, tailing factor ≤ 2, and resolution > 2 (Table 2).

#### 3.2.3. Linearity and Range

**Figure 4:**
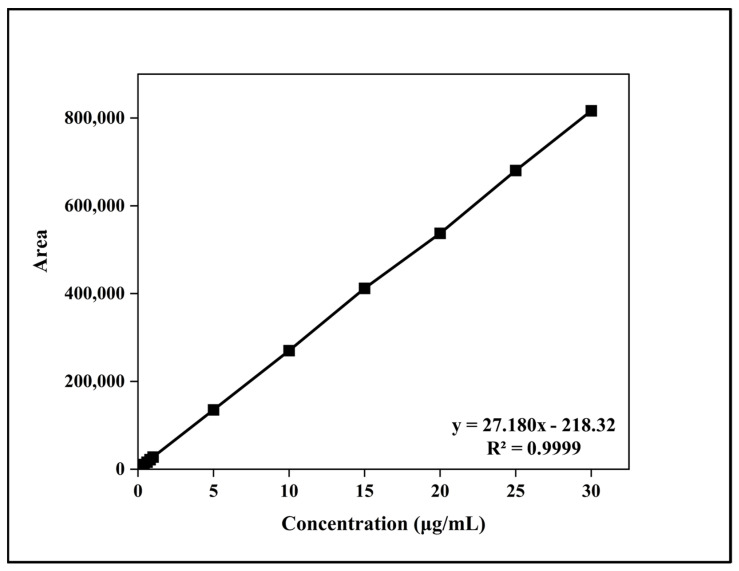
Linearity curve of buserelin acetate (BA) in the concentration range 0.05 µg/mL to 30.00 µg/mL. Chromatographic conditions: C18 column, mobile phase of water:acetonitrile (70:30, *v*/*v*) with 0.035% TFA, pH 2.5; detection at 220 nm, injection volume 10 μL, flow rate 0.8 mL/min.

The linearity of the developed method was established by constructing a calibration curve between peak area and BA concentrations ranging from 0.05 μg/mL to 30.00 μg/mL. A linear relationship was observed across this range, with a correlation coefficient (R^2^) of 0.9999, demonstrating excellent analytical performance (Figure 4). The regression parameters are summarized in Table 3.

### 3.3. Accuracy

The accuracy of the developed RP-HPLC method was tested by adding known amounts of BA solutions at three levels: 1.00 µg/mL, 15.00 µg/mL, and 30.00 µg/mL. The measured values were compared with the actual known amounts to calculate the percentage recovery. The results showed a recovery between 98% and 102%, with an RSD of less than 2%, confirming the accuracy and reliability of the method (Table 4).

### 3.4. Precision

Intraday, interday, and method precision were evaluated by percentage recovery for each set, which was calculated and compared using RSD values, all of which were below 2%, confirming the precision of the method (Table 4).

#### 3.4.1. LOD and LOQ

The LOD and LOQ for BA were found to be 10.00 ng/mL and 40.00 ng/mL, respectively, based on signal-to-noise (S/N) ratios of 3.3:1 and 10:1.

#### 3.4.2. Robustness

The method demonstrated robustness under minor variations in chromatographic conditions, with all RSD values below 2%. These results confirm the method’s consistency and reliability under slightly altered analytical settings (Table 5).

#### 3.4.3. Stability

The stability study confirmed that the standard solutions of BA remained stable for up to 28 days at 4 °C, with no changes observed in peak area or retention time (Table 6).

### 3.5. Ex Vivo Permeation Studies

**Figure 5:**
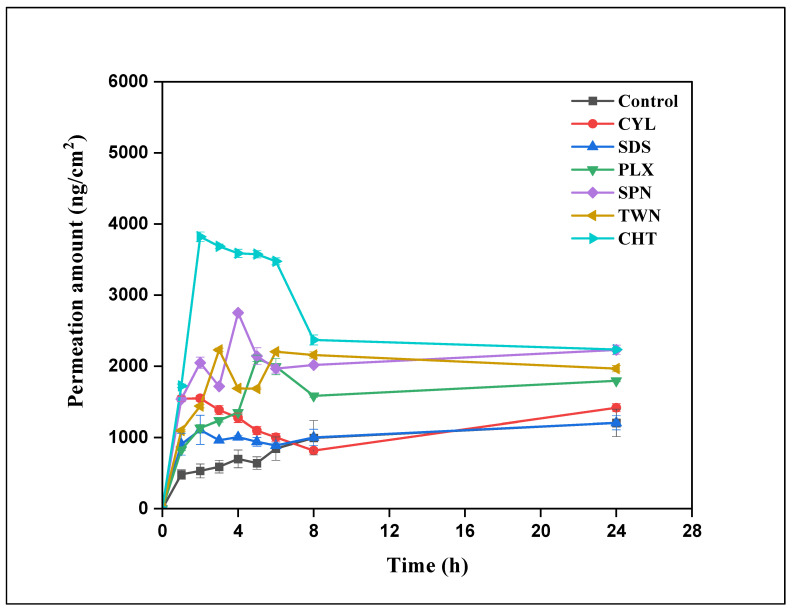
Cumulative permeation profile of buserelin acetate (BA) through porcine vaginal mucosa over 24 h. The experimental groups are Control (BA solution with no enhancer), CYL (2-hydroxypropyl-β-cyclodextrin), SDS (sodium dodecyl sulfate), PLX (Poloxamer 188), SPN (Span 80), TWN (Tween 80), and CHT (chitosan). Data represent mean cumulative permeation values (ng/cm^2^) ± standard error of the mean (SEM) at selected time points (0–24 h).

The validated HPLC method was successfully applied to quantify BA in ex vivo permeation testing. The current study revealed clear differences in BA permeation profiles among the tested PEs, particularly within the critical (0–6) hour window. For the control group, cumulative permeation values at 1, 2, 3, 4, 6, 8, and 24 h were 1320.18 ± 163.41, 1469.59 ± 197.14, 1551.12 ± 228.61, 1767.84 ± 187.08, 1818.61 ± 372.34, 1832.98 ± 390.21, and 2433.75 ± 365.51 ng/cm^2^, respectively (Figure 5). Other enhancers, such as SDS and CYL, demonstrated moderate enhancement, with cumulative permeation values at 6 h of 888.27 ± 35.49 ng/cm^2^ and 1003.13 ± 55.22 ng/cm^2^, respectively. Non-ionic surfactants, including TWN, SPN, and PLX, showed comparatively limited enhancement, with values at 6 h only slightly exceeding that of the control (TWN: 2204.21 ± 13.29 ng/cm^2^, SPN: 1965.39 ± 62.80 ng/cm^2^, PLX: 1994.09 ± 112.44 ng/cm^2^). Although cumulative permeation continued to rise through 8 and 24 h, these later time points primarily confirmed the trends observed during the early phase. The relative permeation enhancement ranking remained consistent across time points: CHT > SDS > CYL > PLX > TWN > SPN.

Among the groups with PEs, CHT demonstrated the highest J, which was statistically higher than other groups. PLX and TWN exhibited comparable fluxes and were not significantly different from BA. Moderate flux values were observed with SPN and SDS, while the lowest flux was recorded with CYL, which was significantly lower than that of most other groups. In summary, the rank order of J (0–24 h) was: CHT > BA > PLX > TWN > SPN > SDS > CYL, with statistically significant differences. In P_app_, CHT again achieved the highest value, which was significantly greater than that of all other groups. BA and PLX showed high P_app_ values, but they were significantly lower than that of CHT. Moderate permeability was recorded for TWN and SPN, whereas SDS and CYL showed the lowest P_app_ values, statistically differing from the higher-performing groups (Table 7).

### 3.6. Kinetic Modelling for Permeation Analysis

The Makoid–Banakar model demonstrated the best fit for both the pure BA and CHT group. For pure BA, the Makoid–Banakar model exhibited an adjusted R^2^ of 0.95, the lowest AIC value, and the highest MSC, indicating superior model performance and supporting the presence of a non-Fickian (anomalous) diffusion mechanism. Similarly, for the CHT group, the Makoid–Banakar model produced a satisfactory adjusted R^2^, AIC, and MSC, further supporting the complex permeation mechanism facilitated by chitosan (Table 8).

## 4. Discussion

This study presents the successful development and validation of a robust and reproducible RP-HPLC method for the quantification of BA, a decapeptide GnRH analogue, with a particular focus on its application in ex vivo permeation studies using porcine vaginal mucosa. A C18-bonded silica column was selected as the stationary phase, since RP-HPLC is among the cutting-edge methods for peptide separation by hydrophobic interactions [27]. Given BA’s polar peptide nature and basic residues, an acidic mobile phase was essential to enhance retention time and improve peak shape. A water:acetonitrile mobile phase containing TFA was used to decrease the ionization of BA and act as an ion-pairing agent, thereby reducing tailing and silanol interactions [18,19]. Detection of BA at 220 nm was selected to monitor BA, as peptides showed strong absorbance in the low ultraviolet range due to the presence of peptide bonds [27]. The robustness of the method under varied flow rates, mobile phase compositions, and column temperatures confirmed its reliability under routine laboratory conditions.

The permeation profile of pure BA (control group) through porcine vaginal mucosa exhibited an initial rapid uptake followed by a marked plateau and a modest rate increase. By 24 h, a further increase in permeation was observed, implying that slow, continued transport occurred after the initial plateau. This trend suggests a non-linear diffusion behaviour, with the plateau phase likely resulting from the combined influence of epithelial barrier resistance, tight junction constraints, and possible BA binding or degradation within the tissue. The modest rise at 24 h may reflect slow release from a drug reservoir effect within the mucosa. Physiologically, these findings imply that BA’s transport across the vaginal mucosa is limited by more than simple diffusion. Buserelin is a hydrophilic decapeptide (~1.2 kDa) [28], which inherently diffuses slowly across lipid-rich biological barriers [4].

Among the six permeation enhancers tested, the 2-hydroxypropyl-β-cyclodextrin (CYL) group exhibited the lowest flux (J) and apparent permeability (P_app_) relative to the control, and the early-time cumulative amount was reduced, indicating limited BA transport across the vaginal mucosa. Cyclodextrins increase apparent solubility, but only free and uncomplexed drug crosses the membrane. When complexation is strong, the free-drug activity decreases and can flatten the permeation profile. According to the cyclodextrin transport model, when diffusion through the aqueous unstirred water layer (UWL) adjacent to the membrane is rate-limiting, CYL can initially control the apparent permeation process [29,30].

Despite being a potent anionic surfactant, the sodium dodecyl sulfate (SDS) group also showed low J and P_app_. In epithelial monolayers, SDS causes concentration-dependent barrier perturbation in mucosal surface, and then suboptimal concentrations can increase diffusional resistance rather than facilitate peptide diffusion, which is consistent with our permeation findings [31]. Additionally, the critical micelle concentration (CMC) of SDS decreases in the presence of salts, meaning effective micellisation may occur at lower nominal concentrations in ionic media such as simulated vaginal fluid; micellar sequestration at higher surfactant levels can further reduce the free drug available for diffusion, ultimately reducing the permeability of drugs [31,32]. Another surfactant, Poloxamer 188 (PLX), showed early cumulative values slightly above those of the control group, followed by a plateau. Mechanistically, PLX primarily stabilises stressed membranes and improves spreading rather than disrupting tight junctions, which is consistent with the modest permeation observed [33,34]. Recently, in vivo, in situ, and molecular data also suggested minimal impact of PLX on tight-junction protein expression at typical formulation levels [35,36].

Additionally, Span-80 (SPN) and Tween-80 (TWN) are both non-ionic surfactants, and each showed moderate permeability compared to the control. SPN forms vesicular aggregates with fluid, flexible interfaces that interact with membrane lipid domains [37]; to our knowledge, evidence that SPN directly opens tight junctions in mucosal epithelium has not been demonstrated. By contrast, TWN primarily reduces interfacial tension and improves microsolubilisation, with limited impact on paracellular pathways [38]. Taken together, studies suggested that surfactants can affect tight-junction integrity, but the mechanisms are not fully elucidated [31].

Compared with the aforementioned permeation enhancers (PEs), chitosan emerged as the most effective enhancer of BA delivery through the porcine vaginal mucosa. In addition, chitosan demonstrated the highest enhancement in both J and P_app_ of BA across porcine vaginal mucosa. This is consistent with its well-established role as a PE due to its mucoadhesive and cationic properties, which facilitate intimate interaction with negatively charged mucosal surfaces, thereby prolonging residence time and increasing drug absorption [39]. In addition, chitosan is known to transiently open tight junctions between epithelial cells by interacting with membrane phospholipids, leading to a reversible reduction in transepithelial electrical resistance and enhanced paracellular transport, especially of hydrophilic macromolecules such as peptides [40,41].

Our findings are further supported by Sandri et al. [42], who demonstrated that 5-methyl-pyrrolidinone chitosan significantly enhanced the vaginal delivery of an antiviral peptide compared to other chitosan derivatives. This formulation not only improved permeation but also exhibited strong mucoadhesive properties, suggesting dual benefits in mucosal drug delivery. The pronounced early enhancement seen with chitosan in our study (i.e., highest cumulative permeation within 6 h) thus aligns well with the literature on vaginal mucosal penetration and peptide transport mechanisms. Our findings also support the notion that chitosan-based systems are especially effective for mucosal peptide delivery.

The kinetic modelling results endorsed this interpretation of a complex and non-Fickian diffusion mechanism. The Makoid–Banakar model, characterised by its combined exponential and power-law structure, provided the best fit to the experimental data (R^2^ = 0.99), accurately representing the biphasic permeation profile. Its low AIC and high MSC values further confirmed its optimal balance between goodness-of-fit and model simplicity [26]. This model’s success reflects its flexibility in describing sigmoidal or biphasic release kinetics. The observed plateau in permeation indicates that after an initial peptide penetration, the mucosal tissue substantially restricts further diffusion, likely due to the peptide’s molecular size, polarity, and possible binding or enzymatic degradation within the tissue. This observation is consistent with the general understanding that mucosal epithelia present a substantial barrier to macromolecules, often requiring specialized delivery approaches to achieve significant absorption [43].

Collectively, the validated RP-HPLC method and ex vivo permeation results offer valuable insight into the development of peptide-based vaginal drug delivery systems. Chitosan, in particular, shows great potential for enhancing mucosal absorption of hydrophilic macromolecules like BA. These findings support the continued exploration of chitosan-based intravaginal platforms in both veterinary and human medicine. Additionally, the robust analytical method provides a reliable tool for future formulation screening, pharmacokinetic evaluation, and translational application of peptide therapeutics in mucosal delivery contexts.

## 5. Conclusions

This study established a robust and validated RP-HPLC method for the quantification of BA, compliant with ICH Q2 (R2) guidelines and capable of accurate detection in permeation enhancer-containing biological matrices. Applying this method, ex vivo permeation studies using porcine vaginal mucosa demonstrated that BA exhibits a biphasic permeation pattern, with early rapid uptake followed by a plateau phase and modest rate increase. Among the tested enhancers, chitosan significantly improved BA permeation, outperforming all other agents within the critical early absorption window. Kinetic modelling confirmed that BA permeation follows a non-Fickian, anomalous diffusion mechanism best described by the Makoid–Banakar model, reflecting the influence of both diffusional and enhancer-mediated transport dynamics. These findings not only highlight the potential of chitosan-based systems for vaginal peptide delivery but also provide mechanistic insight essential for future formulation strategies. Further in vivo studies are warranted to validate these ex vivo results and advance the development of non-invasive BA therapies for reproductive management in veterinary applications.

## Figures and Tables

**Table 1:** Experimental groups for evaluation of permeation enhancers with buserelin acetate (BA).

Group Code	Group Details
Control	BA solution (no enhancer)
CYL	BA + 2-hydroxypropyl-β-cyclodextrin
SDS	BA + sodium dodecyl sulfate
PLX	BA + Poloxamer 188
SPN	BA + Span 80
TWN	BA + Tween 80
CHT	BA + Chitosan

**Table 2:** System suitability parameters for the proposed RP-HPLC method used to quantify buserelin acetate (BA).

Parameters	Specifications	BA Value
Retention time (min)		4.77
Retention time (%RSD)	≤2.0	0.05
Plates	>2000	38,359
HETP		26.11
Areas (%RSD)	≤2.0	0.78
Tailing	≤2.0	1.12
Resolution (Rs)	>2.0	3.29

**Table 3:** Regression and validation parameters for the proposed chromatographic method for the determination of buserelin acetate (BA).

Standard Curve	Range (µg/mL)	Slope	y-Intercept	Correlation Coefficient (R^2^)	Standard Error of the Slope	Standard Error of Intercept
Day 1	0.05–30	27.18	−218.32	0.9999	0.06	867.27
Day 2	0.05–30	27.43	−259.99	0.9999	0.08	1153.69
Day 3	0.05–30	27.40	−217.87	0.9999	0.07	958.29

**Table 4:** Accuracy and precision of the developed chromatographic method at three concentration levels of buserelin acetate (BA). Results are expressed as mean recovery (%) ± RSD (*n* = 6) for accuracy, intraday precision, and interday precision.

Concentrations (µg/mL)	Accuracy (%)	Intraday Precision (%)	Interday Precision (%)
1	101.89 ± 1.38	100.99 ± 1.13	99.37 ± 0.93
15	101.47 ± 0.10	100.90 ± 0.23	101.59 ± 0.23
30	100.03 ± 0.11	99.70 ± 0.13	99.82 ± 0.04

**Table 5:** Evaluation of method robustness under varying chromatographic conditions, including column temperature, mobile phase composition, and flow rate. Recovery values are expressed as mean recovery (%) ± RSD (*n* = 6), demonstrating the reliability of the method under deliberate changes in analytical parameters.

Parameters	Level	Mean Recovery (%)
Temperature (°C)	25	98.43 ± 0.37
	30	99.58 ± 0.92
	35	98.05 ± 0.68

Water:Acetonitrile	68:32	96.59 ± 0.21
	70:30	99.58 ± 0.92
	72:28	95.65 ± 0.07

Flow rate (mL/min)	0.70	98.96 ± 0.34
	0.80	99.58 ± 0.92
	0.90	100.75 ± 0.13

**Table 6:** Stability assessment of the proposed chromatographic method over 28 days. Recovery values are expressed as mean ± RSD (*n* = 3).

Parameters	Day 1	Day 7	Day 14	Day 28
Mean recovery (%)	100.40	100.17	97.79	97.23
RSD	0.39	0.24	0.18	0.14

**Table 7:** Effect of different permeation enhancers on flux (J, ×10^−2^ µg/cm^2^·h) and apparent permeability coefficient (P_app_, ×10^−5^ cm/h) of buserelin acetate (BA) across porcine vaginal mucosa, evaluated using an ex vivo Franz diffusion cell model. Results are expressed as mean ± standard error (SE), based on experiments (*n* = 3). Abbreviations: CYL, cyclodextrin; SDS, sodium dodecyl sulfate; PLX, Poloxamer 188; SPN, Span 80; TWN, Tween 80; CHT, chitosan.

Permeation Parameters	BA	CYL	SDS	PLX	SPN	TWN	CHT
J _(**x**_10^−2^_)_ (µg/cm^2^·h)	0.47 ± 0.14 ^ab^	0.06 ± 0.04 ^d^	0.15 ± 0.02 ^d^	0.43 ± 0.01 ^abc^	0.21 ± 0.02 ^bcd^	0.31 ± 0.03 ^bcd^	0.64 ± 0.03 ^a^
P_app_ _(**x**_10^−5^_)_ (cm/h)	9.40 ± 2.80 ^b^	1.25 ± 0.87 ^c^	3.15 ± 0.581 ^cd^	8.71 ± 0.26 ^bc^	4.25 ± 0.50 ^bcd^	06.21 ± 0.64 ^bcd^	16.20 ± 0.84 ^a^

^a,b,c,d^ Superscript letters indicate statistical groupings according to Tukey’s HSD post hoc test (*p* < 0.05); groups sharing the same letter are not significantly different.

**Table 8:** Mathematical kinetic modelling parameters of ex vivo permeation studies for buserelin acetate (BA) and chitosan (CHT) group.

Model Name	Parameter	BA	CHT
Zero-order	R^2^ adjusted	0.416	0.53
	AIC	128.85	136.22
	MSC	1.21	1.61
Higuchi	R^2^ adjusted	0.82	0.36
	AIC	110.06	129.21
	MSC	0.87	0.735
Makoid–Banakar	R^2^ adjusted	0.95	0.94
	AIC	99.41	111.32
	MSC	2.05	1.50
Peppas–Sahlin	R^2^ adjusted	0.95	0.92
	AIC	99.65	114.25
	MSC	2.03	1.32

## Data Availability

The data supporting the findings of this study are available from the corresponding author upon justified request.
